# Longitudinal Analysis of Latvian Child Growth: Anthropometric Parameters Dynamics from Birth to Adolescence

**DOI:** 10.3390/children11040426

**Published:** 2024-04-03

**Authors:** Silvija Umbraško, Liene Martinsone-Berzkalne, Liana Plavina, Vinita Cauce, Edgars Edelmers, Aleksandrs Starikovs, Janis Vetra

**Affiliations:** 1 Institute of Anatomy and Anthropology, Rīga Stradiņš University, LV-1010 Riga, Latvia; silvija.umbrasko@rsu.lv (S.U.); liene.martinsone-berzkalne@rsu.lv (L.M.-B.); liana.plavina@rsu.lv (L.P.); 054016@rsu.edu.lv (A.S.); janis.vetra@rsu.lv (J.V.); 2 Statistics Unit, Rīga Stradiņš University, LV-1010 Riga, Latvia; vinita.cauce@rsu.lv

**Keywords:** children, physical development, longitudinal study, accelerated growth, anthropology

## Abstract

This study provides a comprehensive analysis of the physical development patterns from birth to adolescence, utilizing a longitudinal dataset of 70 children monitored from birth until 17 years of age. The research focuses on the variability of growth trajectories, emphasizing the role of genetic and environmental factors in influencing these patterns. Key findings indicate that most children undergo one or two periods of accelerated growth, with significant variability in the timing and magnitude of these growth spurts. The study also highlights the adaptive nature of growth changes over generations, influenced by ecological, nutritional, and socio-economic conditions. The longitudinal approach reveals critical insights into the timing of peak growth velocities, demonstrating that girls reach their growth peak approximately one year earlier than boys. The analysis of intergenerational growth patterns suggests a significant increase in average height over the century, attributed to genetic diversity and changes in lifestyle and nutrition. This study’s findings emphasize the importance of updating physical development standards regularly to reflect the changing genetic and environmental landscape. The variability in growth patterns and their correlation with health outcomes in later life highlights the need for targeted public health strategies that address the underlying socio-economic and environmental determinants of health. This research contributes to the understanding of physical development trajectories and provides a foundation for future studies aimed at optimizing health outcomes from early childhood through adolescence. The primary objective of this article is to meticulously analyze the dynamics of height growth and accurately identify the periods of accelerated bodily development within the context of longitudinal research.

## 1. Introduction

Human growth and development are characterized by a complex and non-linear progression, with each life stage presenting unique anatomical and physiological features. This development is defined by variations in body height and mass, along with the pace at which these changes occur, culminating annually. The peak of the growth trajectory of this growth trajectory within a given year is known as the peak growth velocity (PGV), signifying the highest rate of growth achieved [1]. Insights from Aberberga-Augškalne (2001) [1,2] demonstrate that accelerated growth phases are common, with 75% of girls and 74% of boys experiencing at least one such phase, whereas 22% of girls and 24% of boys undergo two and a minority witness even three phases of rapid development. The temporal disparities in growth rates typically span 1.5 years for girls and 1–2.5 years for boys.

Longitudinal studies, by their design, track a cohort of individuals of similar age and background over extended periods, allowing researchers to capture and analyze developmental changes without exerting any influence on the variables of interest. This method of research, inherently correlational, permits a comprehensive examination of growth dynamics across various dimensions [3,4].

Our investigation employs a longitudinal framework to meticulously explore the primary physical development indicator—height—in a cohort of 70 children from birth until the age of 17. This inquiry not only adheres to empirical research methodologies but also endeavors to shed light on the nuances of height variation throughout childhood and adolescence [3].

The changes in physical traits across generations highlight how populations adapt to changing environments, including shifts in ecology, society, nutrition, lifestyle, and health, over the past century [5]. The intersection of genetic predispositions with environmental factors—including dietary habits, physical activity levels, socio-economic pressures, and exposure to infectious diseases—plays a pivotal role in shaping the growth patterns observed in contemporary societies.

The onset of the 21st century has seen significant socio-economic upheavals, such as the economic downturns and mass migration events in Latvia and beyond, which have invariably influenced human anthropological traits [6]. These alterations underscore the importance of regularly updating physical development norms to accommodate the dynamic interplay between genetic evolution and environmental shifts. The lack of consensus among scientists regarding the precise timing and frequency of accelerated growth phases in children highlights the complexity of human development [7]. Furthermore, the balance of physical activity during these critical growth spurts remains essential, underscoring the need for judicious management of sports and physical exertions to support healthy development.

For the first time, a 17-year study is being conducted in Latvia, during which the same children are being surveyed and measured from birth to 17 years of age. Unfortunately, the COVID-19 epidemic prevented this research from being carried out in full. The children we surveyed were 15 and 16 years old in these two years (2021 and 2022). Measurement of these children was limited.

Throughout the 17-year duration of this study, Latvia experienced multiple shifts in its socio-economic landscape, including a significant economic downturn from 2008 to 2010 and the COVID-19 pandemic from 2020 to 2022, which not only disrupted the scheduled measurements but also further deteriorated the socio-economic conditions, and a pronounced migration wave marked by a substantial exodus of people from the country, leading to a demographic crisis. These factors collectively exerted a detrimental effect on Latvia’s birth rate. Data from the Central Statistical Office illustrate a fluctuation in the total fertility rate, indicative of the broader challenges facing the nation [8].

Biological age reflects the degree of maturity of the organism at the relevant age, chronological age—the age of a person according to birth data. However, for many individuals, the level of development can fluctuate within a relatively large amplitude, so we are talking about early, fast-developing children and children with developmental delays. In these cases, the biological age does not match the chronological age. Between these ages, there are particularly large differences during puberty [6].

As the child grows, the growth rate of the body gradually decreases, but this process proceeds unevenly. The child grows most intensively in the first year of life (body mass triples, height increases by 50%). In a study in Lithuania in 2004, 1705 boys and 1576 girls born alive, newborns of 37–42 weeks gestational age, were analyzed. The average body weight for girls was 3454 g, and for boys, it was 3589 g. The average body height for boys was 52.8, and for girls, it was 52.2 cm [9].

Anthropometric data of infants in Latvia are described in a longitudinal study in Riga conducted in 1996. The study was conducted by Krūmiņa, Kokare, and Čivča from the Department of Anatomy and Histology, Faculty of Medicine, University of Latvia [10]. This study lasted until the children under study reached the age of one year.

In the study conducted by the previously mentioned researchers, it is observed that male infants exhibit their most rapid phase of growth during the initial month post-birth, achieving an average height increase of 4.4 cm. Throughout the first six months of life, this growth momentum continues, albeit at a slightly reduced pace, with boys attaining an average monthly height increment of 3.0 cm. In the latter half of their first year, the rate of growth further decelerates, resulting in an average monthly increase of 1.2 cm. Collectively, over the span of their inaugural year, boys experience a cumulative growth of approximately 25.6 cm, highlighting the pace of physical development during early infancy.

Girls grow most intensively in the first month (on average 4.3 cm). During the first 6 months, the average growth is 2.9 cm per month, but in the second half of the year, boys grow 1.2 cm per month. The average growth for girls in the first year of life is 24.7 cm.

The early childhood group includes children aged 1 to 3 years. There is a characteristic decrease in growth rate. In the second year of life, children grow by an average of 10 cm, and their body weight increases by an average of 2.5 kg. In the third year, the child grows by 7.5 cm, and the body weight increases by an average of 2 kg. At the age of one year, a child has a relatively large head, a round face, and short legs [10].

In the studies of other authors, from birth to 1 year of age, the body height of children increases by an average of 25 cm in the first year of life. From the age of two until puberty, there is a relatively constant slow rate of growth. The growth curve varies depending on many factors such as ethnicity, geopolitical conditions, sex, etc. [11].

Early childhood is a critical period of physical and cognitive development that lays the foundation for future well-being. Physical growth includes the attainment of full height and appropriate weight and the enlargement of all organs (except the lymphatic system). Growth from birth to adolescence has two distinct phases:

- Phase one (from birth to about 1 to 2 years of age): This is the phase of rapid growth, although the rate decreases during this period.
- Phase two (from about 2 years to the onset of puberty): In this phase, growth occurs with a relatively constant annual increase [12].

The physical development of infants and children has long been recognized as an important indicator of health and well-being.

The rate of linear growth slows from infancy to early childhood, reaching a relatively constant growth rate of 6 cm/year by age 4–5 years, with little difference between boys and girls. Height growth remains stable until middle childhood, about 5 cm/year between the ages of 7 and 11 [13].

Middle childhood between the ages of 4 and 7 is characterized by a slight growth rate increase of 0.5 cm/year for boys and 0.3 cm/year for girls. However, studies have not observed a specific mid-childhood growth rate, possibly due to the lack of longitudinal, regular studies of growth rates in preschool and school-aged children [14,15].

A 2008 study found that slightly more than half of 579 children had increased height in middle childhood. Pronounced sex differences were evident both in rates of accelerated growth rate (79% of boys compared to 36% of girls) and age (4.8–6.3 years for boys and 3.9–4.8 years for girls) [16]. Sex differences were also observed in a group of Korean children, where the average age of accelerated growth for girls was recorded at 8 years and for boys at 10 years, which is significantly later than other children but still two years before the peak growth rate of puberty. In other studies, the growth rate was quite small, leading some scientists to question its clinical significance [17].

Similar to early childhood, growth during middle childhood progresses slowly. This stage is crucial for cognitive development and social learning. It features a significant cognitive reorganization known as the five-to-seven transition, during which improvements are seen in perceptual abilities, fine motor control, and reasoning. Furthermore, both early (2–6 years) and middle (7–11 years) childhood stages are pivotal for influencing long-term health and functional outcomes. These periods are marked by transformations in body proportions and composition, accelerated brain development and maturation, and hormonal changes [18].

During this period, intersexual differences in growth appear. Boys are, on average, 2 cm taller than girls at age 7, but girls become 1 cm taller by age 10 due to faster growth rates that continue until puberty. The proportionality of the body also changes significantly during this period [19].

Linear growth and BMI trajectories in childhood are associated with the development of several different diseases in adulthood. In a longitudinal study, a large height difference was observed in 9- and 11-year-old children, who were also observed to be obese by the age of 18, indicating accelerated physical maturation [20]. Such mechanisms may also underlie the regularity between obesity and the association of greater maximum height with an increased risk of cancer in adulthood, which has been observed in several studies. Among British women, an accelerated growth rate was found at ages 4–7 and 11–15, which was a risk factor for the development of breast cancer [9]. Similarly, Danish girls who were overweight or tall at ages 7 and 13 had an increased risk of ovarian cancer compared with girls who were not tall or not overweight at those ages [21].

In a study of newborns in Vellore, India, results indicate that children with a higher linear growth rate (height gain) during childhood had a higher risk of developing cardiovascular disease [22].

Although the precise mechanisms underlying the association between faster linear growth in childhood and increased cardio-metabolic and cancer risk are not known, total study findings suggest that the rate of maturation, which affects both linear growth and body mass, may be particularly important health factors in adulthood.

Late childhood begins with an accelerated onset of growth and continues into the ‘teenage’ years. A noticeable growth spurt is probably the most visible sign, along with the appearance of secondary sex characteristics during puberty. Puberty refers to the phase of development in which a child reaches reproductive capacity and spans roughly the first half of adolescence.

The growth patterns of middle childhood will generally differ from the average growth curve, closely related to the characteristics of longitudinal and cross-sectional growth charts.

At this age, boys can grow more than 11 cm per year, and girls can grow more than 9 cm per year. Age at onset and peak of growth spurts are useful reference points for studying the timing and duration of spurts in children [23]. After reaching a peak, the growth rate declines rapidly, indicating the end of the growth cycle near the end of growth, which occurs at about 16–17 years for girls and about 18–19 years for boys in Western countries [23]. Age growth can continue up to twenty years, but height growth will be small (below 1 cm per year) [23]. There are large differences between nations, between individuals, and between both sexes in terms of the height reached at each age, the time of accelerated growth, and the age at which the growth ceases [23].

Children with the same height at birth who are developing faster reach their final height earlier, i.e., stop growing earlier. Children who develop late, on the other hand, reach adult size later and are shorter than the average adult. The effect of differences in the growth rate on the height achieved increases with age and is more pronounced during periods when the slope of the growth curve is steeper, such as adolescence [24].

Thus, the rate differences affect height and growth rates during childhood and adolescence but not adult height [25]. Longitudinal research has consistently demonstrated minimal or no correlation between the timing of puberty in children and their ultimate adult stature, indicating that, on average, children experiencing early, average, or late maturation achieve comparable adult heights, irrespective of intersexual differences.

The shorter growth cycle in early children is offset by a slightly faster growth rate in childhood and a more intense growth spurt in adolescence. The opposite effect is observed in children with late maturation, for whom the growth in childhood is longer but less intense [25].

Variations in body height between adult males and females emerge during adolescence. This period marks a phase wherein the previously slower growth rate of boys during childhood plays a significant role in establishing sex differences in stature observed in adulthood [25].

By the end of this period, most children may also reach their final or adult height size and may surpass their parents in height if there is a positive secular trend.

The age, duration, and length of growth spurts vary greatly among nations and among individuals within the same nation.

In adolescence, with the increased release of sex hormones in the blood, functional changes in the functioning of almost all body systems, characteristic of the puberty period, begin. It is during adolescence that the peculiarities of the hormone balance can cause harmonic or disharmonic acceleration, as well as retardation [26].

Growth in length, especially the growth of long bones, is promoted by somatotropic hormone. A positive correlation between the amount of somatotropic hormone in the blood and the intensity of height growth, especially during puberty, has been proved. This picture is further strengthened by the involvement of male sex hormones—androgens—in the puberty jump [27].

The accelerated growth characteristic of puberty masks the commencement of growth cessation; the more substantially an individual’s height increases, the longer the duration of the puberty period persists until the gonads become fully active. This period’s duration is crucial in explaining the intersex variations in adult height, as boys tend to grow at a slower pace than girls before puberty but continue growing for a longer period. Additionally, boys experience a more prolonged puberty growth spurt, which underpins the observed differences in adult stature between the sexes. At the end of puberty, both boys and girls appear to have sharp sex differences in body proportions. For girls, longer torso, shorter arms and legs, narrower shoulders, and wider pelvis [25].

The proportions of individual parts of the body change with rapid skeletal growth, rapid height growth, strong muscle growth, and development in boys, causing temporary disproportions in body development [21].

Girls aged 16–20 years old are considered young people, and boys aged are considered young people. The beginning of the mentioned age corresponds to the post-puberty phase when the transition of the organism from teenager to adult ends. The contrast between accelerated and delayed youth is striking. Adults are those who are closer to maturity, i.e., those who have accelerated (with rapid growth in their development). Delayed young people are those who develop more slowly, and puberty ends at a young age [27].

## 2. Materials and Methods

For the research project in question, we secured written consent from the parents or legal guardians of the children involved, affirming their voluntary agreement to participate. Furthermore, a formal agreement was established with the director of the maternity hospital, ensuring institutional cooperation and support. It is imperative to emphasize that participation in this study was entirely voluntary, underpinned by informed consent principles. In adherence to contemporary ethical standards and regulations, we have subsequently obtained approval from the Research Ethics Committee for the dissemination of findings related to this project.

In our methodology, the protocol for measuring the physical development of children and adolescents was meticulously designed to ensure consistency and accuracy over the duration of the study. Participants were measured annually, coinciding with the week of their birthday, to ascertain growth metrics when they were precisely a year older.

For the initial two years, measurements were conducted in a horizontal position utilizing a Siber-Hegner Ltd. (Zurich, Switzerland) liberometer of Swiss origin, calibrated and certified for precision. Starting from the age of three, height assessments transitioned to an upright position, employing a Siber-Hegner Ltd. Martin anthropometer, similarly certified and calibrated, adhering to the established methodologies of Martin (1928) [28]. To maintain uniformity across all data collection points, both initial and subsequent measurements were performed by the same experienced professional, assisted by a colleague responsible for recording the data in a detailed questionnaire. This procedure was conducted by a certified specialist with medical training who remains an integral part of our research team.

Initial measurements were seamlessly integrated into the workflow of the labor ward, coordinated with the consent and cooperation of both the parents and the ward management. Following this, subsequent measurements took place in the specialized anthropology laboratory at the Institute of Anatomy and Anthropology, Riga Stradiņš University. Parental consent was obtained well in advance of each measurement session, and appointments were scheduled before the child’s birthday to ensure timely data collection. All assessments were systematically conducted in the morning, before 14:00, to standardize the conditions under which height was measured.

Throughout the course of the study, we experienced a decline in the number of participants due to factors such as emigration and voluntary withdrawal. Consequently, the cohort size has been reduced to approximately 70 participants. Given the unpredictable nature of participation, it remains uncertain whether all enrolled children will complete the measurement sessions up to the age of 19. To honor the commitment of participants reaching this milestone, we present them with a certificate of appreciation, commemorative gifts, and a summary of their growth parameters as documented throughout the study. This gesture has been warmly received, creating a memorable experience for the young participants.

As of this writing, our focus has been on the group of children who have not yet reached the age of 17, selecting a balanced sample of 35 boys and 35 girls who have attained this age to analyze and report on their growth trajectories within the context of our longitudinal study.

Additionally, the body mass index (BMI) was calculated to assess the physical condition of respondents [29].

The obtained data were collected and statistically analyzed using descriptive and inferential statistical methods. In the study, the descriptive representation indicators are minimum values, maximum values, and average values, and the mode is also used, which is the most frequently occurring value or values in the data set—the value of the characteristic whose absolute frequency is the highest. Standard deviation (Std. Deviation) characterizes the deviation from the average, an indicator of data dispersion. Data static processing program SPSS (v.27).

## 3. Results

The research encompassed 70 participants, comprising an equal distribution of 35 boys and 35 girls. At birth, the average height of the boys was reported as 51.1 ± 2.1 cm, with a range extending from a minimum of 47.0 cm to a maximum of 56.0 cm. The girls, on the other hand, had an initial average height of 50.5 ± 1.6 cm, with their heights spanning from 47.4 cm to 53.9 cm, leading to a variance of 5.5 cm for girls and 9.0 cm for boys.

By the age of one, the average height for girls had increased to 75.7 ± 2.4 cm, ranging from 70.0 cm to 79.8 cm. The boys’ average height at this age slightly surpassed that of the girls, standing at 77.1 cm, with the minimum and maximum heights recorded at 72.3 cm and 82.0 cm, respectively, resulting in a height variance of 7.4 cm for boys. Detailed descriptive statistics for the height measurements across both genders at these stages are methodically compiled in Table 1 (for girls) and Table 2 (for boys).

The number of 15- and 16-year-old teenagers is small, so the data are not statistically reliable (due to measurement limitations due to the pandemic).

For girls at the age of 17, the average body height is 170.1 ± 5.3 cm, max. 175.0 cm and min. 156.7 cm. For boys, the average body height at 17 years is 180.8 ± 6.3 cm, min. 168.7 cm and max. 192.4 cm.

**Figure 1:**
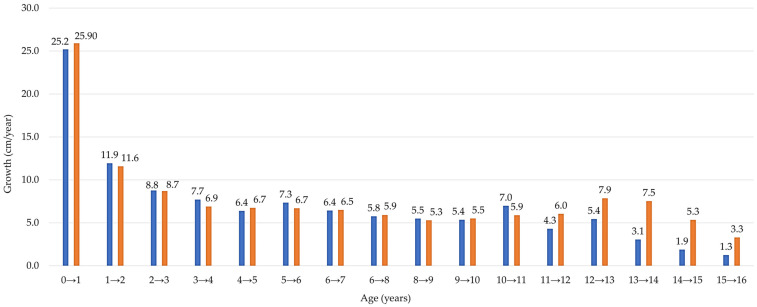
Annual increase in body height by age and sex. The bar chart displays the mean height increment per year (cm/year) along the x-axis, with age groups (years) on the y-axis for boys (orange) and girls (blue).

The total increase in height from birth to the age of 17 is 119.6 cm for girls and 129.7 cm for boys. The first period of accelerated growth in girls is observed at the age of 10–11 years, with an average annual growth of 7.0 cm, but the maximum growth is 7.3 cm/year at the age of 11 to 12 years. In boys, the maximum period of accelerated growth is observed from 12 to 13 years of age with an average growth of 8.0 cm/year, the second period with a growth rate of 7.7 cm/year from 13 to 14 years (Figure 1). The greatest height variation is observed in girls aged 12 years, with a difference between maximum and minimum height of 33.4 cm and 11-year-olds 32.1 cm. For boys, the biggest difference between the maximum and the minimum is observed in 13-year-olds 33.6 cm and 14-year-olds 33.0 cm.

The greatest growth in boys is 25.9 cm from birth to one year; the growth decreases with each subsequent year, reaching the lowest value before puberty of 5.3 cm between nine and ten years. The increase in repeated growth of body height in boys begins at the first stage of puberty.

For girls, the average height at the age of one year is 75.7 ± 2.4 cm, min. 70.0 cm, max. ± 79.8 cm. The average height in 1-year-old boys is 77.1 cm, max. 82.0 cm and min. 72.3. ± 7.4 cm. The descriptive statistics of height are shown for boys (Table 2) and girls (Table 1).

As we can see in Table 3, from birth to the age of 15, the average growth for girls is 115.8 cm, and for boys, it is 122.8. Body length dimensions are characterized by accelerated and decelerated growth periods, which characterize the course of the growth process well. It can be seen that boys grow more evenly after the highest growth in the first year of life. There are two plateaus in growth—from 3 to 7 years, the growth is from 5.9 cm, 6.8 cm, 6.7 cm, 6.6 cm, and from seven to eleven years, 5.9 cm, 5.3 cm, 5.5 cm, and 5.8 cm per year. The acceleration phase of the pubertal growth spurt for boys is noted between the ages of 12 and 14 years, where the annual increase in height averages 7.9 cm/year, accounting for 6.4% of boys in this age bracket, and subsequently averages 7.5 cm/year, representing 6.1% of boys within these age ranges. Among the 35 boys, the greatest annual growth from 12 to 13 years, or the first year of accelerated growth, 40% of the boys had an annual growth of more than 9 cm/year, and 57% of them had an increase in height of more than 10 cm/year. The largest increase is 13.4 cm. In the second year of accelerated growth, 10 of the 31 measured boys have an annual growth of more than 9 cm/year.

Compared to WHO (World Health Organization) data from 5 to 14 years of age, body height by age ranges from 15 to 85 percentiles for boys. At the age of five, 11.4% of boys’ height is below 105.5 cm (15th percentiles), 17.1% over 115.0 cm (85th percentile), and 71.5% of boys have height between 15th and 85th percentile (Table 4). From 72.7% to 60%, the height of 5- to 17-year-old boys is within the norm according to the standards proposed by the WHO.

In girls, the acceleration phase of the spurt is observed between eleven and twelve years of age, lasting one year, compared to boys, when the growth is 7.3 cm (6.26%). Between the ages of eleven and twelve, 48.5% of girls grew taller by more than 7 cm/year. The biggest increase is 10.5 cm/year.

Table 5 shows the distribution of girls’ body height by age (5–14 years) from the 15th to the 85th percentile. More than 55% of girls aged 5 to 14 are in the 15th to 85th percentile, which, compared to WHO data, coincides with the proposed standards or norms for the respective age.

More than a third of 9-year-old boys (35.2%) grow faster than other boys of this age, as well as a third of boys in the 10-year-old (31.4%) and 13-year-old (34.2%) age groups grow faster. Slower growth is observed in boys at the age of 5 (11.4%), 12 years (5.7%), 13 years (5.7%), and 14 years (6.5%) boys. One can conclude that three growth variants are observed in the growth process of boys—normal, accelerated, and lesser slow growth.

More than half of girls’ body height is within the normal range by percentiles, compared to the standards proposed by the WHO for ages 5 to 14 years. More than a third of girls have faster growth in the age stages of 8 years (31.4%), 11 years (31.4%), and 12 years (37.1%). On average, about 3% of girls between the ages of 5 and 13 grow according to the slow growth type.

Comparing the dynamics of growth parameters for boys and girls, it is found that the maximum period of accelerated growth for boys is from 12 to 14 years and for girls from 11 to 12 years.

In our research, the body mass index (BMI) of participating children was assessed, and the findings predominantly fell within the normal range. However, an individualized examination revealed that four boys and six girls exhibited an elevated BMI (Table 6).

## 4. Discussion

The evolutionary transformation observed across centuries, marked by an augmented body stature and an earlier onset of puberty, stands as a pivotal phenomenon within contemporary human biology. This trend not only holds profound implications for the fields of medicine, education, and society at large but also serves as a barometer for the health and well-being of children and adolescents. The rate of growth and body length are critical indicators, reflecting the health status of youths within specific age brackets. Standards derived from the physical assessments of homogeneous child cohorts are instrumental for educational institutions and healthcare professionals, aiding in the precise evaluation of individual physical development.

The socio-economic shifts, patterns of migration, the prevalence of sedentary lifestyles, variations in nutritional quality, environmental pollution, and other contributing factors collectively forge an environment that precipitates morphological changes within the population. An augmentation in body height is often heralded as a signifier of enhanced living standards, whereas a diminution is indicative of declining conditions [30].

In Latvia, comprehensive longitudinal studies spanning the 20th and early 21st centuries have meticulously documented the physical, sexual development, and postural attributes of children and adolescents. Notably, research conducted by Feder (1933) [31] between 1930 and 1933 identified the peak growth rates in Latvian children, with boys aged 14–15 experiencing an annual increase of 7.2 cm and girls aged 12–13 observing an annual growth of 6.3 cm. Miller’s (1962) [32] findings in 1962 further corroborated these observations, pinpointing the maximal growth increments at similar ages. A comparative analysis with early 20th-century studies from Britain, Scandinavia, and Poland, among others, reveals a nuanced variation in growth patterns across different geographies. For instance, between 1880 and 1950, the average height increment for children aged 5 to 7 was approximately 1 cm and about 2.5 cm for adolescents. In specific locales such as Stuttgart and Leipzig, [33] notable increases in average height were recorded between the years 1913 to 1937 and 1918 to 1930, respectively, highlighting regional disparities in growth trends. These insights underscore the intricate interplay between genetic, environmental, and socio-economic factors in shaping the physical development trajectories of populations across the globe [33].

Several researchers have documented that during puberty, boys can experience growth spurts exceeding 11 cm per year, while girls may see growth rates of up to 9 cm per year, highlighting significant variances across nations, individuals, and sexes [23]. This variability in growth patterns underscores the complex interplay of genetic, environmental, and hormonal factors that influence physical development. It is known that children of identical birth lengths can exhibit diverse growth trajectories—categorized as early, middle, and late development stages. These variations in growth rates significantly impact height and growth velocity during childhood and adolescence; however, they do not ultimately affect the final adult height. Consequently, studies, including those by Sheffler (2018), reveal no direct correlation between the timing of puberty and the final stature achieved in adulthood [25].

Umbraško’s (2003) comprehensive cross-sectional analysis, spanning 1998 to 2003 and involving 1700 children aged 7–18 years, elucidated two distinct periods of accelerated growth in boys: the initial phase occurring between 8 and 9 years with an average increase of 7.7 cm per year, followed by a second phase around 12–13 years with a growth rate of 7.9 cm annually. This research indicated that boys experienced a total height increment of 55.1 cm from ages 7 to 17. Conversely, girls displayed their first significant growth spurt between 10 and 11 years, with an annual height increase of 6.7 cm and a subsequent spurt from 11 to 12 years at a rate of 7.3 cm. For girls, the cumulative height gain from ages 7 to 17 amounted to 42.8 cm. Both genders ceased growing by the age of 17, with the study also examining postural developments, finding that 34.5% of 17-year-old boys (average height of 180.4 ± 5.5 cm) and 41.5% of girls (average height of 167.6 ± 6.0 cm) exhibited postural disturbances, including scoliosis and daily back pain, with 69.0% diagnosed with round-concave posture [6].

Duļevska’s (1997) research, conducted between 1996 and 1997 and surveying 1350 school-aged girls, focused on physical and sexual development, identifying the pivotal period of accelerated growth in girls as occurring between 11 and 12 years, with an annual height increase of 7.8 cm. This period notably aligns with the emergence of secondary sexual characteristics and the initiation of puberty. Duļevska’s (1997) findings also suggest a notable shift in body proportions over the century, with shoulder width increasing and hip width decreasing among girls in Riga, indicative of significant morphological changes over time [34].

At the onset of the 21st century, the study conducted by Cederštrēma (2007) between 2005 and 2007, encompassing the physical and sexual development of 1359 school-age boys, highlighted a significant phase of accelerated growth occurring between 13 and 14 years of age, with an extraordinary annual height increment of 8.5 cm. This surge in growth represents the most substantial increase ever recorded within the Latvian cohort, surpassing the growth rates identified in preceding studies. According to Cederštrēma’s (2007) research, adolescents ceased their growth at 18 years, achieving an average stature of 180.8 ± 6.3 cm. Notably, the timing of this accelerated growth in boys aligns precisely with the manifestation of secondary sexual characteristics, as outlined in Cederštrēma’s (2007) findings [35].

When juxtaposed with the findings of prior research, it is evident that girls in Latvia enter puberty approximately 1.5 to 2.0 years earlier than their male counterparts. Our comprehensive longitudinal study corroborates this developmental timeline, pinpointing the apex of accelerated growth in girls between the ages of 11 and 12 years, marked by an annual increase of 7.2 cm, whereas boys experience this peak growth phase at 12–13 years, with a rate of 7.8 cm. Historical data, such as that from Feder’s (1936) [31] study in 1936, reveals that the average height of 17-year-old girls was once 170.0 cm, which then escalated to 174.6 cm in Miller’s (1963) [32] findings, and further to 180.4 cm in Umbraško’s (2005) [6] study. In our longitudinal analysis, the stature of 17-year-old boys reached 180.8 cm, indicating a remarkable increase of 10.8 cm over the last 90 years in Latvia. Similarly, the average height for 17-year-old girls showed a progressive rise from 160.3 cm in Feder’s study to 162.6 cm in both Miller’s (1963) [32] and Umbraško’s (2005) [6] research, culminating at 166.3 cm in our study. This gradual increment of 6.0 cm over nine decades underscores a consistent trend of physical growth among Latvian girls, reflecting broader shifts in public health, nutrition, and living conditions over the course of the 20th and early 21st centuries.

When examining average body heights across various nations through cross-sectional analyses conducted in the late 20th century, our findings align with those reported by researchers in other countries. In Estonia, an accelerated growth phase in boys between 12 and 13 years of age results in an average annual height increase of 7.8 cm, ref. [36] culminating in an average body length of 181.4 cm for 17-year-old boys. Polish adolescents exhibit two distinct growth spurts: the first occurring between ages 9 and 10 with a growth rate of 5.4 cm per year, followed by a second surge between ages 13 and 14, witnessing a rate of 7.9 cm per year [37].

Notably, there appears to be a lack of studies within Latvia focusing on adolescents who may experience health issues as a result of accelerated growth phases. Such periods of rapid development are critically sensitive times in a child’s life. It has been observed that the availability of sports clubs within schools has diminished, with many activities being either costly or beyond the financial reach of parents, leading to either a lack of physical activity or, conversely, physical overload in children who participate in multiple sports clubs daily. This situation is further exacerbated by other adverse factors, including unhealthy and insufficient dietary practices, deteriorating socio-economic conditions, mass immigration, and other variables that collectively impact the general health status of the population. Our ongoing study intends to extend until the participants reach the age of 19, offering further insights into these phenomena and their implications on physical development and health.

One significant challenge is the attrition rate experienced over the course of the longitudinal research. A notable number of participants have emigrated or elected not to continue their involvement, leading to a reduction in the cohort size. This dropout rate introduces potential biases into the findings, as the remaining sample may not adequately represent the initial diversity of the population, both in terms of genetics and environmental influences.

## 5. Conclusions

Our research pinpoints a crucial phase of maximum growth in height happening from 11 to 12 years in girls and from 12 to 13 years in boys, emphasizing the sex-specific beginnings of growth spurts. The results indicate that, on average, girls reach their peak growth velocity around one year before boys, suggesting an earlier onset of puberty and its related growth spurts in females. Such earlier maturation in girls necessitates specific considerations in terms of physical, psychological, and educational support.

Most children, representing over half of both sexes, display a standard or average pace of bodily growth and development across all examined ages. However, about one-third of the study group shows signs of accelerated or premature development, whereas slower growth rates are observed less frequently or are absent at certain developmental stages, reflecting a wide range of growth speeds within the studied population.

The study notes no significant sex differences in height from ages 1 to 10 years. Yet, a change is observed at 11 and 12 years, with girls temporarily exceeding boys in height before boys overtake girls in stature starting from age 13. This period highlights the interaction between biological maturation and growth trends. In girls, two key periods of rapid growth were identified, starting at ages 5–6 and again at 11–12, whereas boys undergo three periods of accelerated growth, beginning at ages 4–5, 12–13, and extending into 13–14. These insights deepen our understanding of sex- intersexual growth patterns.

Over the course of this longitudinal study, the pattern of physical development observed among participants reflects findings from cross-sectional research conducted in the early 21st century. Relative to growth metrics from the 20th century, the average height of 17-year-old boys has increased by 10.8 cm and for girls by 6.0 cm, indicating that the onset of puberty-related growth spurts now occurs approximately one year earlier, illustrating that the growth trajectories of children in the 21st century have accelerated compared to those observed in the previous century. This intergenerational increase in growth suggests a shift in developmental timelines, potentially mirroring changes in environmental, dietary, and socio-economic factors influencing the health and growth of modern youth.

## Figures and Tables

**Table 1:** Descriptive statistics of the girls’ body height (cm).

Sex	Age	Average	SD	Minimum	Maximum
Girls	0	50.5	1.6	47.4	53.9
	1	75.7	2.4	70.0	79.8
	2	87.6	2.8	81.4	94.4
	3	96.4	3.5	88.4	103.3
	4	104.1	3.8	95.1	112.5
	5	110.5	4.3	100.6	121.3
	6	117.8	4.4	108.0	128.3
	7	124.3	5.0	113.8	135.5
	8	130.0	5.0	120.0	142.5
	9	135.5	5.5	124.7	147.2
	10	141.5	6.2	130.0	155.6
	11	148.5	7.2	134.0	166.1
	12	155.8	7.5	140.0	173.4
	13	161.4	6.3	148.8	176.1
	14	164.4	5.8	153.7	178.0
	15	163.8	6.2	152.6	178.0
	16	169.1	5.7	160.9	178.9
	17	166.3	5.4	156.7	175.0

**Table 2:** Descriptive statistics of the boys’ body height (cm).

Sex	Age	Average	SD	Minimum	Maximum
Boys	0	51.1	2.1	47.0	56.0
	1	77.1	2.4	72.3	82.0
	2	88.8	3.1	82.3	94.6
	3	97.5	3.9	90.6	105.8
	4	104.4	4.1	97.3	111.0
	5	111.2	4.3	110.6	118.8
	6	117.9	4.3	110.6	126.4
	7	124.5	4.8	117.1	133.5
	8	130.4	5.2	121.9	140.7
	9	135.6	5.6	126.4	147.4
	10	141.1	5.7	131.5	153.6
	11	147.0	6.7	135.2	162.3
	12	153.2	7.2	140.6	169.8
	13	161.1	8.3	143.4	177.0
	14	168.8	8.6	149.0	182.0
	15	176.8	5.4	166.5	183.4
	16	175.0	6.9	164.5	185.9
	17	180.8	6.4	168.7	192.4

**Table 3:** Absolute and relative average height increase in boys and girls.

Age (Years)	Girls		Boys	
	Growth (cm)	Percent (%)	Growth (cm)	Percent (%)
0–1	25.2	21.8	25.9	21.1
1–2	11.9	10.3	11.7	9.5
2–3	8.8	7.6	8.7	7.1
3–4	7.7	6.7	6.9	5.6
4–5	6.4	5.5	6.8	5.6
5–6	7.4	6.3	6.7	5.5
6–7	6.4	5.6	6.6	5.3
7–8	5.8	5.0	5.9	4.8
8–9	5.5	4.7	5.3	4.3
9–10	6.0	5.2	5.5	4.5
10–11	7.0	6.0	5.8	4.8
11–12	7.3	6.3	6.2	5.1
12–13	5.6	4.9	7.9	6.4
13–14	3.1	2.6	7.5	6.1
14–15	1.9	1.6	5.4	4.4
Total	115.8	100	122.8	100

**Table 4:** Distribution of body height in boys by WHO standards (%).

Age (Years)	WHO Standards 15–85% (Body Height cm)	Percentiles		
		<15%	15–85%	>85%
5	105.5–115.0	11.4	71.5	17.1
6	110.0–121.1		71.5	28.5
7	116.3–127.2		65.8	34.2
8	121.4–133.1		65.8	34.2
9	126.3–138.8		64.8	35.2
10	131.2–144.4		68.6	31.4
11	132.0–150.1		71.5	28.5
12	141.7–156.4	5.7	65.7	28.5
13	148.2–163.7	5.7	60.0	34.2
14	155.2–175.8	6.5	67.7	25.8

**Table 5:** Distribution of girls’ height by WHO standards (%).

Age (Years)	WHO Standards 15–85% (Body Height cm)	Percentiles		
		<15%	15–85%	>85%
5	104.7–114.5	5.7	74.3	20.0
6	109.8–120.4	2.8	68.6	28.5
7	115.1–126.5	2.8	68.6	28.5
8	120.5–132.6	2.8	65.8	31.4
9	126.2–138.8	2.8	68.6	28.5
10	132.0–145.3	5.7	68.5	25.7
11	138.1–151.9	2.8	65.8	31.4
12	144.1–158.3	5.7	57.2	37.1
13	149.2–163.9	2.9	61.9	35.2
14	152.6–167.0		73.5	26.4

**Table 6:** Overview of average body mass index (BMI) trends for both boys and girls, spanning from birth through 14 years of age.

Age (Years)	Boys	SD	Girls	SD
0	13.9	1.20	13.7	1.23
1	17.7	1.47	16.6	1.28
2	16.9	1.25	15.9	1.25
3	16.5	1.20	15.7	0.96
4	16.1	1.09	15.5	0.80
5	15.9	1.38	15.6	1.05
6	16.1	1.64	15.3	0.95
7	16.7	2.14	15.7	1.25
8	17.2	2.37	16.1	1.52
9	17.9	2.41	16.7	1.71
10	18.5	2.65	17.2	2.02
11	19.1	3.12	18.1	2.51
12	19.7	3.33	18.7	2.48
13	20.3	3.84	19.4	2.46
14	21.5	4.26	20.1	2.21

## Data Availability

The data presented in this study are available on request from the corresponding author due to ethical reasons.
